# A qualitative investigation of LGBTQ+ young people’s experiences and perceptions of self-managing their mental health

**DOI:** 10.1007/s00787-021-01783-w

**Published:** 2021-04-26

**Authors:** Rosa Town, Daniel Hayes, Peter Fonagy, Emily Stapley

**Affiliations:** 1 Faculty of Brain Sciences, University College London, London, UK; 2 The Evidence Based Practice Unit, Anna Freud National Centre for Children and Families, London, UK

**Keywords:** LGBTQ+ young people, Self-management, Self-care, Sexual minority adolescents, COVID-19 lockdown, Youth mental health

## Abstract

There is evidence that young people generally self-manage their mental health using self-care strategies, coping methods and other self-management techniques, which may better meet their needs or be preferable to attending specialist mental health services. LGBTQ+ young people are more likely than their peers to experience a mental health difficulty and may be less likely to draw on specialist support due to fears of discrimination. However, little is known about LGBTQ+ young people’s experiences and perceptions of self-managing their mental health. Using a multimodal qualitative design, 20 LGBTQ+ young people participated in a telephone interview or an online focus group. A semi-structured schedule was employed to address the research questions, which focussed on LGBTQ+ young people’s experiences and perceptions of self-managing their mental health, what they perceived to stop or help them to self-manage and any perceived challenges to self-management specifically relating to being LGBTQ+ . Reflexive thematic analysis yielded three key themes: (1) self-management strategies and process, (2) barriers to self-management and (3) facilitators to self-management. Participants’ most frequently mentioned self-management strategy was ‘speaking to or meeting up with friends or a partner’. Both barriers and facilitators to self-management were identified which participants perceived to relate to LGBTQ+ identity. Social support, LGBTQ+ youth groups and community support were identified as key facilitators to participants’ self-management of their mental health, which merits further investigation in future research. These findings also have important implications for policy and intervention development concerning LGBTQ+ young people’s mental health.

## Introduction

Self-management has been defined as, “[t]he taking of responsibility for one's own behaviour and wellbeing” [1, p. 1]. There is a lack of conceptual clarity in the self-management literature, as evidenced by multiple conflicting definitions to describe the concept [2]. For example, one study of chronic disease in older adults argued there is a distinction between ‘self-care’ as preventative and ‘self-management’ as managing the impact of a current difficulty or disease [3]. However, in mental health, as probably in many other long-term conditions, this distinction does not hold up, with interventions involving elements of self-management and self-care being used preventatively to identify and manage the early warning signs of manic episodes in bipolar disorder [4]. A recent study highlights the crossover between self-management and self-care in youth mental health by detailing strategies, such as meditation or deep breathing, which could be used for both illness prevention and management of existing symptoms [5]. Thus, it is likely that the terms self-management and self-care in the context of mental health are not mutually exclusive and may lie on a continuum of techniques and strategies.

In the last few decades, self-management has expanded from long-term illness toward youth mental health. The idea of caring for or managing oneself is appearing with increasing frequency in published research [5, 6], United Kingdom (UK) policy [7, 8] and reports from charities and health bodies [9, 10]. This may be due to rising societal awareness of the heightened prevalence of mental health difficulties in young people in the UK (NHS Digital, 2018) and long wait times to access specialist youth mental health support [11]. In turn, attempts to facilitate the use of alternative or additional support options which better meet the needs of young people, are person-centred and exist in the places they ordinarily go have also increased in recent years [12]. Indeed, young people themselves have said that the efficacy of self-help and self-management resources, approaches and techniques should be a top research priority in relation to youth mental health interventions and services [13].

In young people’s mental health, self-management strategies could include self-care approaches [e.g., 5], unguided self-help interventions [e.g., 14] and coping or emotion regulation strategies [6]. For young people, strategies that can be employed on one’s own have been described as non-professionally mediated interventions [5]. Despite burgeoning interest in this area, there is a dearth of research investigating the nuances of self-management in young people’s mental health, particularly for young people whose difficulties may not be chronic, are undiagnosed or are below clinical thresholds. This group may be of particular importance if self-care happens to differentiate those who remain sub-threshold from those who are diagnosable and seek professional help. Such investigations are warranted to clarify the concept of self-management and understand what young people perceive they are doing to self-manage their mental health, which in turn will enable self-management strategies recommended to young people to be evidence-informed and better meet their needs.

In looking at the success (or otherwise) of health management strategies at a population level, it makes sense to look at groups at particularly high risk. Among socially excluded groups, such as lesbian, gay, bisexual, transgender and queer (LGBTQ+) young people, there is a higher prevalence of mental health difficulties [15]. One in three LGBTQ+ young people in the UK will experience a mental health difficulty [15], and this figure is greater than the one in eight young people in the general population who will experience a mental health difficulty [15]. Evidence suggests that sexual minority adolescents are also more likely than their heterosexual peers to experience high levels of depressive symptoms, self-harm, lower life satisfaction and lower self-esteem [16]. The minority stress model posits the higher prevalence of mental health difficulties in the LGBTQ+ community is due to their experience of hostile social environments fuelled by prejudice, discrimination and stigma [17].

When a difficulty is encountered, there is evidence that LGBTQ+ people are less likely to access health services due to fear of discrimination [18], with mental health services perceived to be the most discriminatory amongst health services [19]. LGBTQ+ people also experience higher dissatisfaction with health services than heterosexual people [19]. As they are less likely to access health services, it is possible that LGBTQ+ young people are already using strategies to self-manage their mental health, although to-date, there has been no research into this. It is possible that LGBTQ+ young people are forced to self-manage their mental health due to a desire to avoid stigmatising services, and it may be possible that they may have developed particularly effective and robust strategies which may be useful to learn more about. Further research in this area could particularly benefit a group which is often overlooked, marginalised and for whom the pressures of daily life may be greater than their heterosexual or cisgender peers. This research will also enable us to see if LGBTQ+ young people need additional help in self-management and how this process might be facilitated for them.

Recently, a survey showed that 72% of LGBTQ+ young people in Northern Ireland use the Internet and social networks as a source of information or support [20], suggesting the potential utility of these formats for the distribution of information about self-management. However, a review of qualitative research related to LGBTQ+ young people’s mental health highlighted that many LGBTQ+ young people feel they need more support from their school, community and mental health providers as well as more information [21]. It is also not known what factors stop or help LGBTQ+ young people to self-manage their mental health. Research into this area is needed to ensure that the strategies and techniques LGBTQ+ young people are using and being recommended to self-manage their mental health are safe, evidence-based and have a positive impact on mental health outcomes.

To better understand the helpfulness of self-management strategies for LGBTQ+ young people’s mental health, and to potentially facilitate LGBTQ+ young people’s self-management of their mental health, there is a need to investigate which strategies LGBTQ+ young people are already using and those that they find helpful, as well as the perceived barriers and facilitators to successful self-management of their mental health. The current study attempts to address this gap in the literature and highlight the self-management process from the perspective of LGBTQ+ young people to learn more about specific factors affecting self-management of mental health for this group.

## Research questions

1. What are LGBTQ+ young people’s experiences and opinions of using strategies or techniques to self-manage their mental health?
2. What are LGBTQ+ young people’s perceptions of what stops them from or helps them to self-manage their mental health?
3. What are LGBTQ+ young people’s perceptions of specific challenges (if any) for LGBTQ+ young people in self-managing their mental health?

## Methods

### Participants

#### Recruitment and sampling strategy

During the two-month sampling period, young people from diverse ages, ethnicities, gender identities, sexual orientations and geographical locations in the UK (including major metropolitan areas and rural areas, as well as areas known to have a significant LGBTQ+ presence and those not known for this) were recruited. The primary researcher contacted organisations specialising in gender diversity as well as general LGBTQ+ youth groups. Collecting these data and involving LGBTQ+ people in research can help to send a signal that their views and preferences are taken seriously and valued [18].

On the basis of accessing a wide range of views, a total of 85 LGBTQ+ youth groups or associated organisations, 12 LGBTQ+ University Societies, three post-graduate student cohorts and one participation group were identified via a Google search and the primary researcher’s existing organisational contacts and invited to participate in this research via email by the primary researcher. Staff from 40 interested groups were asked to distribute expression of interest forms to the young people they worked with along with a brief description of the project.

Following guidelines for reflexive thematic analysis, a sample size determined by saturation was not established before starting data collection [22]. Saturation is “the point at which no new information, codes or themes are yielded from data” [23, p. 2]. Diversity of the sample and richness of the data were monitored during sampling. The decision to stop sampling at 20 participants was based on three considerations: sufficient diversity of the sample, quality or richness of the data being collected in relation to the research questions and the practical capacity of the primary researcher.

#### Demographic information

Of the 20 young people who participated in this study, their ages ranged from 13 to 24 years (*M* = 19.30, SD = 3.37).

In terms of gender identity, 12 participants identified as female, four preferred to self-describe, three identified as male and one preferred not to say. Self-described gender identities included nonbinary, gender queer and asexual. A total of 13 participants stated that their gender identity was the same as the sex assigned to them at birth, six as not the same as the sex assigned to them at birth, and one preferred not to say.

Regarding sexual orientation, eight participants preferred to self-describe, six identified as bi, four as a gay woman/lesbian, one as a gay man and one as heterosexual. Self-described sexual orientations included queer, questioning, pansexual, homoromantic asexual and asexual biromantic.

In terms of ethnicity, 13 participants identified as White—British, two as White—any other white background, one as White—Irish, one as Asian—Indian, one as mixed—White and Asian, one as Mixed—White and Black Caribbean and one as any other ethnic group—Persian.

### Ethical considerations

Ethical approval for this study was obtained from the University College London Research Ethics Committee (Project ID: 17641/001). During the consent process, young people were reminded that they could leave the interview or focus group at any time without giving a reason and still receive a £10 voucher, which was offered to all participants. Parent/carer consent was obtained for participants under the age of 16, which was followed by assent from these participants. Participants were informed that interviews and focus groups would be kept confidential, barring the disclosure of harm to the participant or another person.

### Data collection

A total of 20 young people participated in either an online focus group (*n* = 4 participants across two focus groups), or in a one-to-one telephone interview (*n* = 16 participants) over the one-month data collection period. Interviews and focus groups were conducted remotely via video call or phone owing to COVID-19-related restrictions on in-person contact.

Semi-structured interview and focus group schedules were developed by the primary researcher. This format allowed the researcher to ask open-ended questions to elicit information about participants’ thoughts, feelings and beliefs in relation to self-management [24]. Questions explored participants’ perceptions of the term ‘self-management’, the types of self-management strategies and techniques they perceived themselves to be using, and anything that stopped or helped them to self-manage. There was also an additional question relating to specific challenges experienced by the LGBTQ+ community in terms of self-management.

During the pilot phase of this research, the interview and focus group schedules were modified to include several follow-up questions if a young person mentioned the first COVID-19 lockdown in the UK (beginning in late-March 2020), or any other time-specific period, to investigate how participants’ experiences of self-management may by influenced by time and context. This decision allowed for discussion around the impact of the first COVID-19 lockdown in the UK as and when it came up, as the interviews and focus groups were conducted during this period. A decision was also made during the piloting phase to move solely to conducting individual interviews, rather than focus groups, due to the richness and highly personal nature of the data that was elicited in the interviews.

### Data analysis

#### Thematic analysis

The data were analysed to answer the three research questions following guidelines for reflexive thematic analysis [22] and drawing on the step-by-step process developed by Braun and Clarke [25]. This involved checking the transcripts against the audio files, reading the data in its entirety multiple times and taking notes, creating ‘codes’ which captured interesting aspects of the data systematically across the entire data set, bringing the codes and corresponding data extracts together into initial themes, checking that the themes captured the essence of the data extracts, continuing to refine and analyse each of the themes, and finally producing a report featuring vivid examples from the data extracts for each theme [25].

A second researcher (ES) reviewed the initial coding structure once it had been generated by the primary researcher to check that the codes reflected the content of the included data excerpts. After this, the primary researcher further refined the codes and began to group the codes into themes and subthemes, i.e., overarching categories encompassing all of the included codes. The primary researcher then checked that the codes and included data reflected each of the themes.

#### Epistemological stance

The primary researcher holds a realist ontological and relativist epistemological stance in relation to the analysis of these data. This can be described as a critical realist approach, which asserts that a reality independent from subjective experience exists, while situating the findings of this research in the belief that it is not possible to objectively understand or fully access this reality [26]. The primary researcher also acknowledges that the manner in which participants perceive reality is subjective. In other words, different people interpret reality in different ways, given that “knowledge is always situated” [27, p. 7]. Thus, the analysis focuses primarily on the semantic or language-based themes identified in the dataset, with some investigation of the potential latent meanings of these themes, as well as the wider societal and cultural context. An inductive approach to analysing these data was taken, meaning that the results are data-driven [25].

## Results

### Theme 1: Self-management strategies and process

#### Subtheme 1: List of strategies for self-management

A total of 51 strategies for self-management of mental health were identified. These encompassed strategies participants perceived themselves to have personally used as well as those they perceived others to have used (see Table 1).

**Table 1:** Specific self-management strategies and frequency mentioned

Self-management strategy	Number and percentage of participants who mentioned strategy
Speaking to or meeting up with friends or a partner	13 (65%)
Distraction	9 (45%)
Being productive or achieving something	8 (40%)
Writing or journaling	8 (40%)
Exercise	7 (35%)
Speaking to or meeting up with family	7 (35%)
Watching television, a specific series, or a movie	6 (30%)
Listening to or playing music	6 (30%)
Going to therapy or counselling, speaking to a professional	5 (25%)
Sleeping, napping, or having a sleep schedule	5 (25%)
Participating in enjoyable activities or hobbies	5 (25%)
Balancing emotions or thoughts, recognising thinking errors, or stopping a downward spiral	4 (20%)
Reading	4 (20%)
Separating or detaching yourself from the situation	4 (20%)
Going outside, going for a walk	4 (20%)
Meditation	3 (15%)
Eating well, having a healthy diet	3 (15%)
Playing a game	3 (15%)
Attending an LGBTQ+ youth group	3 (15%)
Taking medication	3 (15%)
Being positive	3 (15%)
Being on your own or having time to yourself	3 (15%)
Having a treat	3 (15%)
Cooking or baking	2 (10%)
Volunteering or helping others	2 (10%)
Having a shower, taking a bath, hygiene	2 (10%)
Taking a break, taking the pressure off	2 (10%)
Drinking enough water	2 (10%)
Painting and drawing	2 (10%)
Gardening	2 (10%)
Breathing	2 (10%)
Drinking alcohol	2 (10%)
Applying therapeutic techniques	2 (10%)
Mindfulness	2 (10%)
Speaking to a cuddly toy	1 (5%)
Peer support	1 (5%)
Letting it happen or breaking down	1 (5%)
Recording a letter to yourself	1 (5%)
Actively doing something	1 (5%)
Using apps	1 (5%)
Getting dressed in the morning	1 (5%)
Taking vitamin D tablets	1 (5%)
Stimming	1 (5%)
Putting on comfy clothes	1 (5%)
Self-harm	1 (5%)
Crafting	1 (5%)
Repetitive handwashing	1 (5%)
Yoga	1 (5%)
Positive self-reminders, mantras, or setting intentions	1 (5%)
Anchoring techniques	1 (5%)
Martial arts	1 (5%)
Using resources	1 (5%)
Using drugs or substances	1 (5%)
Target shooting	1 (5%)

Participants’ most frequently mentioned self-management strategy was ‘speaking to or meeting up with friends or a partner’. This dovetails with the perceived importance of social support, which was identified as a facilitator to self-management. While this strategy involved someone other than the ‘self’, participants described how they self-initiated the help-seeking behaviour of reaching out to others for support with their mental health.

*Having people speak to you about their own mental health can be very reassuring, ‘cause like helping someone does that, it makes you feel like helping yourself with your own mental health is a lot [more] feasible.* (Interviewee 4)

Many of the strategies described by participants involved elements of balancing, distracting oneself from, or regulating thoughts or emotions through the process of participating in the self-management activity or strategy. For example, with regards to target shooting as a self-management strategy, one participant explained:

*It's more about distracting yourself, letting your body cool down, and then, when you've cooled down, then you can have that rationalised, proportionate response.* (Interviewee 7)

#### Subtheme 2: Awareness, reaction and prevention

Participants described a process of awareness, reaction and prevention in self-managing their mental health. In terms of awareness, participants highlighted the importance of noticing or paying attention to signs of good or deteriorating mental health.

*Even if not actively working on figuring it out, just passively paying some mind to consider what kind of things are good for your mental health.* (Interviewee 4)

In terms of reacting, participants described actively using self-management tools, strategies, techniques or skills to combat difficulties or problems they were experiencing, which could help them when they were feeling overwhelmed. In terms of prevention, being proactive and vigilant were also perceived to be important aspects of the self-management of participants’ mental health, even when they were not experiencing poor mental health.

*So the proactive things I do are from a place where nothing bad is necessarily happening, and my mental health is not really flaring up or, or doing anything, um, particularly bad, or, or, particularly abnormal, but I aim to keep it that way by doing things in anticipation.* (Interviewee 2)

### Theme 2: Barriers to self-management

#### Subtheme 1: Self-management can be hard work

Nearly all participants (*n* = 18) described times when they felt too low, tired or not in the right mindset to self-manage their mental health, which could sometimes be exacerbated by experiencing mental illness or chronic pain. They also explained that sometimes attempts to self-manage could be unsuccessful, which could lead to feelings of discouragement or disappointment.

*It can be very disheartening if you think, if it seems like you’re putting in all this effort and nothing’s coming of it.* (Focus Group 2, Participant 1)

The actual process of self-management was described as tiring and time-consuming, with participants explaining they sometimes did not know what to do or where to start.

*I feel like one of the biggest inhibitors of self-management is if your emotions get too loud and [if] those thoughts get too loud, it's very hard to try and think over them.* (Interviewee 6)

#### Subtheme 2: Strategy-specific challenges

Participants perceived there to be negative or less helpful factors about some self-management strategies. For instance, regarding speaking to or meeting up with friends, one participant explained:

*Especially if you’re just talking about how you’re having mental health problems to other people who have those problems all the time, it can kind of cause this, like, negative feedback loop um, with that. So that can be less helpful.* (Interviewee 5)

Participants also mentioned self-management strategies or techniques which they had found to be unhelpful, ineffective or counterproductive, such as drinking alcohol, self-harming or repetitively washing their hands.

*For a little period, I self-harmed... I was kind of looking for an outlet anywhere I could find at that moment. And, I'd, I’d never feel better afterwards, I'd feel worse.* (Interviewee 9)

#### Subtheme 3: Wanting to wallow

Participants described feeling like they were sometimes their own worst enemy or wanting to wallow in the negative emotions they were feeling, which could prevent them from engaging in self-management. In this context, the ‘self’ was perceived to be a barrier to self-management.

*If you just can’t bring yourself to do it, then, it, I found that you sort of end up wallowing in like, the sort of self-pity.* (Interviewee 11)

Participants described feeling responsibility to self-manage their mental health. This was perceived to operate as a double-edged sword, both allowing participants to rely on themselves and be decisive, but also creating pressure and a sense that the onus was on them.

*It’s like really hard knowing one day you’re going to have to be the one that is relatively solely responsible for, like, engaging in self-care and managing, like, your wellbeing.* (Focus Group 2, Participant 2)

#### Subtheme 4: Fear of judgement

While participants perceived others to be important in helping them to self-manage their mental health, they also discussed factors which might hinder them from reaching out, such as feeling judged or thinking their family would be worried about them.

*Like if I told [my family] what actually went on, they'd be kicking off and really worried and panicky. So, that isn't helpful.* (Interviewee 7)

On the other hand, having a safe space or feeling safe to talk about their thoughts and emotions with others was viewed as helpful in self-managing participants’ mental health.

*Having good supportive relationships and being in a space where I don’t feel very threatened or have any, any severe issues, uh, means that I can work on myself, uh, a lot better.* (Interviewee 2)

#### Subtheme 5: Cultural and environmental challenges

Participants described ways in which culture and environment could influence self-management. It was highlighted by participants that there are different cultural understandings of self-management, which could cause people to view the concept differently from one another.

*In (Foreign Language 1) it’s not that uh common to use like the word self-management or talk about emotional regulation. It’s not like integrated into the vocabulary.* (Focus Group 1, Participant 1)

Participants also highlighted that some cultures have more challenges around accessing treatment, a different understanding of mental health, or a perception that mental illness is taboo. These challenges could also apply to certain generations within a particular culture, or over time as a culture became more progressive.

*And, and I think in (Country 1), it’s just kind of taboo, like, in not, not so much anymore, but it’s just impossible to even, like, if you Google ‘mental health’, like, there will be fewer results.* (Interviewee 12)

A perception was also highlighted that participants’ physical location could influence their ability to self-manage their mental health, particularly in environments not conducive to certain self-management strategies or techniques.

*It depends on the environment you’re in. ‘Cause if you’re in, like, an education-based building in the middle of the day, it’s not like you can go to your bedroom and try and relax or whatever.* (Interviewee 13)

A number of perceived challenges to self-management of mental health stemming from the COVID-19 lockdown in the UK were also discussed by participants. Participants perceived these challenges to negatively affect their ability to self-manage as well as damaging to their general mental health and wellbeing. They described reverting to behaviours indicative of poor mental health like repetitive handwashing, feeling anxiety about going outside, experiencing a lack of structure or routine, not being able to attend locations where they could participate in self-management strategies or enjoyable activities due to closures, not having a reason to get out of the house or to get out of bed, not being able to attend LGBTQ+ youth groups, experiencing an extension of waiting lists or difficulties in accessing mental health care, not being able to spend as much time with friends, work pressures increasing during the lockdown period, experiencing uncertainty and instability resulting from exams being cancelled and feeling anxiety about the pandemic and the future.

*It was mostly just a very lengthy waiting list. Of course, it was exacerbated by the lockdown.* (Interviewee 2)

#### Subtheme 6: Digital complexities

Participants mentioned a number of digital complexities relating to self-management and self-care. They perceived ‘self-care’ to be the most recognisable term owing to online promotion. Participants explained that this could lead to particular societal connotations of self-care which could be negative or centred around profit.

*It just kind of has become this whole industry of bullet journals and things that I think make finding actual self-care a little complex.* (Interviewee 12)

Participants described researching self-care and self-management online and accessing a ‘plethora of resources’ including online videos, Facebook groups, information about LGBTQ+ groups, information about counselling and professional help, information about different self-management strategies and online message boards.

*So I, kind of, looked online and seeked advice and, you know, through mental health professionals included.* (Interviewee 6)

However, participants did not always find these suggestions or resources helpful, and at times the abundance of available information could be perceived to be overwhelming.

#### Subtheme 7: ‘Outness’ affects self-management

Participants described how an LGBTQ+ person who was not completely out^1^ might find it more challenging to self-manage, as they might have to self-manage on their own. Not being out was perceived to contribute to challenges around accessing therapy for LGBTQ+-related difficulties, not having social or school support around being LGBTQ+, not being able to access an LGBTQ+ youth group and not having access to judgement-free spaces.

*I didn’t feel like I had anyone I could talk to about it, because I didn’t feel comfortable to come out to anyone yet.* (Interviewee 4)

Nearly all participants (*n* = 18) perceived cultural or societal intolerance of LGBTQ+ people to have a profoundly negative impact on LGBTQ+ young people’s ability to self-manage their mental health, which was also influenced by their degree of ‘outness’. Participants discussed how they believed LGBTQ+ people were more likely to experience adverse events or trauma resulting from homophobia, transphobia, discrimination or a general lack of understanding or support from others. These were linked by participants to increased stress, a reduced capacity to cope, and internalised homophobia.

*I have a lot of in-built insecurities that I didn’t experience until I came out, and a lot of sadness and trauma inherited from the community almost. And so, it adds something else to tackle, so it just means you may have more to be contending with.* (Focus Group 2, Participant 2)

Participants described how growing up with intolerance could lead to a detrimental sense of internalised homophobia, which was perceived to contribute to poor mental health and hinder self-management, as it was another challenge to tackle in helping oneself. However, participants also highlighted that the self-management techniques and strategies they had successfully used were not specifically tailored for LGBTQ+ young people, and that there was not necessarily a lack of access to self-management tools and techniques for LGBTQ+ young people.

*I came out, when was it? Like a couple of years ago, um, but that hasn’t significantly, like, a few experiences happened that did make my mental health somewhat worse, but, the techniques that I was using worked just as well for those experiences as they did for others*. (Interviewee 3)

Participants perceived family members’ rejection of their LGBTQ+ identity to have a negative impact on their capacity to self-manage. These stressful or negative experiences could lead to fear or low self-esteem, which participants perceived to undermine their help-seeking efforts.

*Trying to help yourself and self-care, um, it might be difficult in a situation where you're around others that are completely undermining you and, um, are trying to steer you away from trying to help yourself.* (Interviewee 14)

Participants also described how they felt they had to be less open with their parents, carers or particular members of their family due to fears that they did not meet their heteronormative expectations. They feared that they would be met with homophobia, transphobia or other discrimination based on their sexual orientation or gender identity.

*There’s also the other part of my family, which is homophobic, and I’m terrified of coming out to.* (Interviewee 1)

This fear of a negative reaction was cited as something that might prevent participants from drawing on others for support in self-managing their mental health.

### Theme 3: Facilitators to self-management

#### Subtheme 1: Balance and routine

Participants described the importance of feeling balanced and being able to get into a regular routine of self-management. They described mental health in terms of one’s mental state, which ideally would be balanced or controlled. The act of self-management was described as a method for regaining balance which could lead to happiness, focussing on the positive or feeling more calm and clear-minded.

*When I was in the routine of, of meditating, it becomes a habit, and then it becomes a lot easier to do that. And the m-, the easier it becomes to do something, a coping mechanism that helps me, like meditating, the easier it then becomes to do any other given task on that day.* (Interviewee 2)

In relation to location, having a quiet space to self-manage away from others was described as important by participants.

*Some people, I don’t know, don’t have like a quiet space where they can go to kind of relax and, and meditate and kind of feel better in themselves.* (Interviewee 15)

Finally, participants viewed having a routine for self-management as important. This could involve a daily pattern of behaviour, writing things down or planning ahead.

#### Subtheme 2: Intrinsic benefits of self-management

Despite experiencing challenges in self-management, participants also described intrinsic benefits stemming from self-managing their mental health. These included a sense of agency, ownership, freedom and confidence that came from successfully self-managing or knowing how to self-manage.

*You know that you have yourself to thank for it, at the end of the day. Like it feels incredibly good when it does go right to be able to say, kinda yeah, I did that, there’s no two ways around it.* (Focus Group 2, Participant 1)

This ability to manage their mental health was attributed by participants to allowing them to live a better life. Similarly, participants described how the process of self-management could be quite enjoyable, as it could involve activities they already liked to do.

*A lot of those things are, are things that do make me happy anyway. It’s not all just a slog of having to do these things to keep my mental health, you know, working well.* (Interviewee 2)

Participants also described how feeling connected, grounded, present and grateful could contribute to and result from self-managing their mental health. This was intertwined with a sense of perspective, purpose, peace or clarity of mind.

#### Subtheme 3: Importance of social motivation and support

When asked about good mental health, participants described interacting socially with others, including speaking to family and friends, contributing to society and enjoying spending time with friends.

*A lot of my friends have had experiences with mental health issues in the past, so they can, they can, uh, commiserate with that when I want to talk about it. But also just, hanging out and having a good time and laughing and that sort of stuff just makes me feel happy, and that can, that can make the difference between having a, a good day and a bad day, uh mental health wise.* (Interviewee 2)

Conversely, participants highlighted that a sign of poor mental health could be feeling unable to be around other people, isolating oneself, taking others’ comments personally or misjudging social situations.

*If it's, say, a negative self-doubt that you might feel, that's a product of poor mental health, be- say you're out with your friends, and, you know, usually a joke might, th- that you might laugh at, suddenly is now a deep personal attack.* (Interviewee 6)

This participant also went on to explain that their interpretation of a situation when their mental health was poor did not necessarily align with what was happening in reality.

The majority of participants (*n* = 16) perceived social motivation and support to be important in self-managing their mental health. This involved participants’ family members, friends, flatmates or partners, who could be helpful in providing a listening ear, giving encouragement, reminding them to self-manage or checking their emotions or thoughts.

*It makes it much more fun and um, motivational like if there’s other people on board, then you’re like, okay, we’re all in this together.* (Focus Group 1, Participant 2)

#### Subtheme 4: Asking for help and vulnerability

Participants highlighted the perceived importance of being able to ask for help from others or seeking help from a professional to self-manage their mental health. This was linked by participants to good self-management, and it was acknowledged that sometimes outside help was necessary despite efforts to self-manage alone.

*I think sometimes it becomes that idea of, "You can do it by yourself. You don't need somebody else to help self-manage." When sometimes you do need someone else's perspective or someone else's professional skills to give you new ideas or to help you help yourself. *(Interviewee 5)

It was also highlighted that asking for help involved an element of vulnerability and opening up.

*So, self-management also involves the ability to be vulnerable, and the ability to speak to other people about things that you’re going through.* (Focus Group 1, Participant 2)

#### Subtheme 5: Benefits of the COVID-19 lockdown in terms of self-management

Participants highlighted a number of perceived benefits resulting from the COVID-19 lockdown which related to their ability to self-manage. One of these was having more free time to stop, self-reflect and think without distraction, which could help with focussing on self-management.

*Mostly since lockdown, ‘cause obviously I’ve, you know, I’ve had a lot of time to just sort of self-evaluate, self-reflect, and I’ve found that maybe this is something that I was neglecting.* (Interviewee 11)

Other perceived benefits included developing a closer relationship with family members, spending less time commuting, developing a more regular routine, not having to prepare for stressful exams, being more positive and putting things into perspective, appreciating spending time with friends more, having the opportunity to participate in social justice initiatives and striking a better work-life balance.

*Like lockdown has actually been a godsend because I was not looking forward to A Levels and I was getting myself worked up in it, and also my atmosphere at school wasn't great... So, it's now like having more free time, having my dedicated space for like, the meditation.* (Interviewee 7)

#### Subtheme 6: LGBTQ+ community helps with self-management

Participants described a perception that identifying as LGBTQ+ and having access to the wider LGBTQ+ community, either through youth groups, friends who are also LGBTQ+, online resources or forums specifically for LGBTQ+ people or LGBTQ+ events like Pride, could help them to self-manage their mental health.

*Like, there's a solid community that I've been able to access because I identify as LGBTQ*+*... There’s also, kind of, opportunities to reach out to others like you, and kind of reach out to people who can help, with the same perspective. *(Interviewee 9)

In comparison to someone who did not identify as LGBTQ+, participants felt they might experience fewer challenges in self-managing their mental health. They attributed this again to their access to the LGBTQ+ community, which they perceived to strengthen their sense of togetherness and camaraderie (both online and in person) and to provide them with a safe space to talk to others. This was seen as something potentially inaccessible to people who were not members of the LGBTQ+ community.

*And I suppose there’s also ways that it makes it, not easier, but, in other ways more positive, such as having this community, the LGBTQ*+ *community, who understand you, without even having to know you... maybe someone who isn’t LGBTQ*+*, who doesn’t have the best surroundings, may not have that community sense, um, of encouragement.* (Focus Group 2, Participant 1)

## Discussion

This research investigated LGBTQ+ young people’s experiences and perceptions of self-managing their mental health. Specifically, this research aimed to illuminate LGBTQ+ young people’s experiences and opinions of using strategies or techniques to self-manage their mental health, their perceptions of what stops them from or helps them to self-manage their mental health, and their perceptions of specific challenges (if any) for LGBTQ+ young people in self-managing their mental health. Three overarching themes were identified covering self-management strategies and barriers and facilitators to self-management (see Table 2). These themes are discussed in turn and compared and contrasted with existing literature.

**Table 2:** Themes and subthemes

Theme	Subtheme
Self-management strategies and process	List of strategies for self-management
	Awareness, reaction and prevention
Barriers to self-management	Self-management can be hard work
	Strategy-specific challenges
	Wanting to wallow
	Fear of judgment
	Cultural and environmental challenges
	Digital complexities
	‘Outness’ affects self-management
Facilitators to self-management	Balance and routine
	Intrinsic benefits of self-management
	Importance of social motivation and support
	Asking for help and vulnerability
	Benefits of the COVID-19 lockdown in terms of self-management
	LGBTQ+ community helps with self-management

### Specific self-management strategies

Several of the self-management strategies identified in the current study align with previous research investigating coping [6] and non-professionally mediated interventions [5]. Stapley et al. [6] identified similar coping strategies, including ‘digital or media entertainment’, ‘creative activities’, ‘being physically active’, ‘positive thinking or optimism', ‘ignoring people, feelings or situations’, ‘social support’ and ‘other professional support’ (see Table 1). Likewise, a number of non-professionally mediated interventions identified in Wolpert et al.’ study reflected the self-management strategies identified in the current study, including (but not limited to) reading, self-harm, talking to someone you know and trust, positive thinking, physical exercise, sleep, mindfulness, walking, spending time outdoors in nature, warm bath, writing things down and making music [5]. This overlap suggests that participants perceived some coping strategies and non-professionally mediated interventions to also be self-management strategies, giving credence to the idea that these lie on a continuum of caring for oneself.

### Barriers to self-management

LGBTQ+ young people in the current study perceived themselves to be at a higher risk of experiencing trauma or adverse events resulting from identity-related discrimination. This is reflected in research showing that LGBTQ+ young people describe experiencing rejection, isolation, discrimination, abuse, bullying and homophobia or transphobia [21], as well as evidence suggesting that sexual minority adolescents are more likely to experience all forms of bullying and victimisation in comparison to their heterosexual peers [16]. Findings from the current study also suggest that experience of these negative or adverse events can have a detrimental effect on LGBTQ+ young people’s capacity to self-manage their mental health, which aligns with previous literature suggesting that adverse events can affect young people’s ability to cope [e.g., 29]. While the association between adverse events and poorer mental health for LGBTQ+ young people has been established [16], more research is needed into how LGBTQ+ young people’s capacity to self-manage may mitigate this. However, the barriers ‘wanting to wallow’ and ‘self-management is hard work’ in the current study could suggest that even in the absence of adverse events, self-management is a challenging process for LGBTQ+ young people to initiate or engage in at times.

Lack of acceptance from family was also perceived by participants to be a barrier to self-management of mental health. This aligns with the individual and family self-management model, which maintains that family members play a key role in the management of illness, particularly for younger people [30]. It is possible that the degree to which an LGBTQ+ young person is out with their family and friends also acts as a barrier, as an LGBTQ+ young person who is not out may fear judgement from family and friends, which was also mentioned as a barrier to self-management in the current study.

The COVID-19 lockdown in the UK appeared to contribute to a number of perceived barriers to self-management of mental health in the current study. The negative impact of the lockdown on LGBTQ+ young people’s mental health was echoed in research by Kneale and Becares [31], who found high levels of both stress and depressive symptoms amongst LGBTQ+ people during the lockdown, particularly in the case of younger and transgender respondents, as they were more likely than others in the LGBTQ+ community to have experienced some form of discrimination during the pandemic. Experiences of discrimination such as these have been associated with greater symptoms of emotional difficulties like anxiety and depression amongst transgender people [32]. The findings from the current study did not suggest that participants had experienced additional discrimination during the lockdown, but instead that the barriers to self-management resulting from the pandemic were partly perceived to be due to not being able to access vital services, attend LGBTQ+ youth groups, socialise as often with friends or attend extracurricular activities due to closures. These barriers align with the minority stress model, which posits that mental health outcomes for LGBTQ+ people are affected by coping and social support, both from the community and individuals [17], which were likely negatively affected by lockdown-related closures and government-mandated restriction on in-person socialising.

### Facilitators to self-management

The current study suggests that the degree which an LGBTQ+ person is out influences their perceived ability to access self-management support from friends, family or their community. This aligns with the minority stress model, which positions ‘minority identity’ as an important factor influencing LGBTQ+ people’s mental health outcomes, coping and social support [17]. This also links with findings from previous research suggesting that LGBTQ+ young people who are not out might struggle to access self-management support from friends, family or their community [21]. The current study’s findings also suggest that LGBTQ+ young people who have access to the LGBTQ+ community may find it easier to self-manage their mental health, and that this community can serve as an oasis of social support and acceptance in a heteronormative world. This aligns with findings suggesting that LGBTQ+ people perceive the LGBTQ+ community to have a positive effect on their mental health and wellbeing through providing support and reducing a sense of isolation [33]. There is evidence that peer support such as this amongst LGBTQ+ young people can reduce a sense of marginalisation and the likelihood of poor mental health outcomes [34, 35]. Additionally, social support and motivation and having a safe space to self-manage were mentioned by participants in the current study as facilitating self-management of their mental health, which aligns with policy highlighting the importance of having safe and supportive social environments in the mental health of LGBTQ+ young people [36].

Positive effects of the COVID-19 lockdown in terms of self-management, particularly in relation to having more free time and thinking positively, were echoed in findings from the Teenagers’ Experiences of Life in Lockdown (TELL) Study [37]. This research suggested that young people in the UK experienced an enjoyable sense of relief from stressors they were previously experiencing in their daily lives and a sense of positivity during the lockdown [37]. Likewise, the negative implications of the lockdown mentioned in the current study also aligned with those in the TELL Study, including young people feeling increased fear, anxiety and distress about COVID-19 as well as other aspects of their daily lives [37].

Finally, participants in the current study highlighted their tendency to seek out information regarding self-management or self-care online. The influence of digital support on LGBTQ+ young people’s self-management of their mental health merits further exploration in future research, as there is evidence that the Internet is one of the main methods of accessing support for members of the LGBTQ+ community [21]. Therefore, it is likely that an intervention to facilitate self-management would benefit from a digital format, and there is some evidence that young people from a nonclinical population already use digital technology for reducing stress and would find a digital self-management tool useful [38].

### Strengths and limitations

This study has several strengths. Firstly, a diverse group of participants in terms of ethnicity, gender identity, sexual orientation, age and geographic location was recruited, which increased the likelihood that a broad range of views were accessed. Secondly, the inductive nature of the analysis performed allowed for the identification of themes which went beyond the original research questions (e.g., ‘Wanting to wallow’) and spoke to the heterogeneity of experiences regarding LGBTQ+ young people’s self-management of their mental health. A further strength was the enlistment of an additional researcher in checking the coding for the analysis, which enhanced the trustworthiness of the analysis by ensuring that the primary researcher’s interpretations were grounded in the data [39]. Finally, this study involved members of the LGBTQ+ community who identified with any sexual orientation or gender identity, including those who identified as heterosexual, ensuring that a full range of views could be accessed without adhering to rigid or binary conceptualisations of gender identity or sexual orientation. This is important because it emphasised the researchers’ position at the beginning that gender is a construct and reduced the likelihood that the results were influenced by a bias of heteronormativity, which could have led participants to feel less able to talk about their experiences as it this be perceived as discriminatory or naïve.

There are also some limitations to this research. These findings cannot be generalised to all LGBTQ+ young people but may be applicable to wider populations, as many of the experiences and perceptions may also ring true for other young people in the UK. Additionally, while recruitment yielded a good geographic spread of participants, not all areas in the UK were covered (e.g., Scotland), which means that if there is geographic variation in experiences and perceptions in these areas, it may not have been captured by this study. It is also possible that these data were skewed toward young people who were more likely to be out, as the majority of participants were involved with LGBTQ+ youth groups, meaning the views of LGBTQ+ young people who are less out and therefore more likely to draw on anonymous sources of self-management support merit further investigation. Additionally, participants under the age of 16 were required to provide parent/carer consent to participate, which meant that young people under the age of 16 who were not out to their parents may not have chosen not to participate. Although participants 16 and older did not require parental consent, it is likely that young people who were living with parents/carers who were unsupportive of LGBTQ+ people chose not to participate due to fears of being overheard or not having sufficiently private space available to them. Successfully recruited participants were also those who had access to computers, headphones, tablets or mobile phones, potentially excluding young people with reduced financial means. Future research may benefit from recruiting LGBTQ+ young people from the general population, particularly in-person as COVID-19-related restrictions ease, as this might yield greater diversity of views relating to self-management and provide a helpful point of comparison.

## Conclusion

This is the first research study, to our knowledge, to investigate LGBTQ+ young people’s experiences and perceptions of self-managing their mental health, as well as the barriers and facilitators to self-management for this group. Findings established that LGBTQ+ young people perceive themselves to be using multiple strategies to self-manage their mental health and perceive there to be a number of barriers and facilitators to this process. These findings support further exploration into the development of or provision of research-informed support to an intervention or policy to support self-management, particularly that which can be tailored for specific groups (e.g., gender diverse individuals), as some groups might find particular ways of self-managing their mental health more helpful than others [32]. A key area of future investigation should be into social and LGBTQ+ youth group or community support as key facilitators to the self-management of mental health, as these were highlighted by participants in the current study and have implications for policy and intervention development. More research is needed into digital mental health interventions for LGBTQ+ young people, and how such interventions could facilitate LGBTQ+ young people’s self-management of their mental health, to potentially improve mental health outcomes for this group.

## Data Availability

Due to challenges in ensuring the anonymity of qualitative data, these data are not available to other researchers.

## Footnotes

1 The word ‘out’ used by participants is viewed by the primary researcher to refer to ‘coming out’, which can be defined as “To acknowledge or declare openly that one is homosexual. Also in extended use with reference to other sexual or gender identities” [28, p. 1].
